# Dexmedetomidine Versus Fentanyl as an Adjuvant to 0.75% Ropivacaine in Ultrasound-Guided Infraclavicular Brachial Plexus Block: An Observational Study

**DOI:** 10.7759/cureus.114094

**Published:** 2026-08-06

**Authors:** Sumitha Babu, Kagathi Ramesh Vasantha Kumar, Aditi V Prabhu, Shruthi Rajagopala

**Affiliations:** 1 Anaesthesiology, Adichunchanagiri Institute of Medical Sciences, Nagamangala, IND; 2 Anaesthesia and Critical Care, University Hospital of North Tees, Hardwick, GBR; 3 Anaesthesiology, Sri Balaji Medical College Hospital and Research Institute, Renigunta, IND; 4 Critical Care Medicine, Kasturba Medical College, Mangalore, Mangalore, IND

**Keywords:** brachial plexus block, dexmedetomidine, fentanyl, ropivacaine, ultrasonography interventional

## Abstract

Background

Peripheral nerve blockade is increasingly used in upper limb orthopedic surgery to provide effective anesthesia and prolonged postoperative analgesia while reducing systemic opioid requirements. Ultrasound-guided infraclavicular brachial plexus block provides reliable anesthesia for surgeries of the elbow, forearm, wrist, and hand, and adjuvants are frequently added to local anesthetics to improve block quality and prolong analgesia. Dexmedetomidine and fentanyl are two such adjuvants, but their comparative effects on onset, duration, and quality of blockade remain unclear. This observational study compared dexmedetomidine and fentanyl as adjuvants to ropivacaine in ultrasound-guided infraclavicular brachial plexus block.

Methodology

A prospective observational study was conducted in the Department of Anaesthesiology, Adichunchanagiri Institute of Medical Sciences, over 18 months from November 2016 to April 2018. Sixty eligible patients aged 18 to 65 years, of ASA physical status I or II, undergoing elective upper limb orthopedic surgery under ultrasound-guided infraclavicular brachial plexus block were enrolled consecutively and observed across two exposure groups of 30 each, categorized according to the adjuvant selected by the treating anesthesiologist as part of routine clinical practice. Group A received ropivacaine 0.75% (30 mL) with dexmedetomidine 50 micrograms, while Group B received ropivacaine 0.75% (30 mL) with fentanyl 75 micrograms, under ultrasound guidance. Sensory blockade was assessed by the pinprick method and motor blockade by the modified Bromage scale. Heart rate, blood pressure, and oxygen saturation were monitored, and adverse effects were recorded.

Results

Baseline demographic and clinical characteristics were comparable between the two groups, except mean body weight, which was lower in Group A. The mean onset of sensory blockade was 8.45 minutes in Group A compared with 1.83 minutes in Group B, and the mean onset of motor blockade was 11.27 minutes versus 2.87 minutes, both p<0.0001. Complete sensory blockade was attained at 12.90 minutes in Group A versus 5.90 minutes in Group B, and complete motor blockade at 20.12 minutes versus 7.06 minutes, both p<0.0001. Duration of sensory blockade was 883.17 minutes (SD 66.27) in Group A versus 404.00 minutes (SD 95.85) in Group B, and duration of motor blockade was 819.07 minutes versus 380.38 minutes, both p<0.0001. Postoperative analgesia lasted 977.79 minutes (SD 66.90) in Group A compared with 492.66 minutes (SD 106.14) in Group B, p<0.0001. Complete motor blockade was achieved in 24 patients (80%) in Group A versus 16 patients (53.33%) in Group B, p=0.0285. Systolic blood pressure was significantly higher in Group B from five minutes onward, while diastolic pressure was consistently higher in Group A. No hypotension, bradycardia, or respiratory depression was recorded in either group.

Conclusion

Dexmedetomidine, when added to 0.75% ropivacaine for ultrasound-guided infraclavicular brachial plexus block, was associated with a significantly longer duration of sensory and motor blockade and postoperative analgesia, along with denser motor blockade, compared with fentanyl, despite a slower onset. Fentanyl was associated with a faster onset of blockade. Both adjuvants demonstrated a favorable safety profile, favoring dexmedetomidine when prolonged analgesia is clinically prioritized.

## Introduction

Effective perioperative pain control remains a cornerstone of favorable surgical outcomes, and regional anesthesia techniques have progressively supplanted systemic opioid-based analgesia owing to their superior pain control and reduced side-effect burden [1]. The brachial plexus, formed by the ventral rami of C5 through T1, can be blocked at multiple anatomical levels to provide surgical anesthesia for the upper extremity [2]. Among these approaches, the infraclavicular technique targets the cords of the plexus as they encircle the axillary artery beneath the pectoralis minor, offering a stable catheter position and reliable coverage of the elbow, forearm, and hand [3]. First described in the early 20th century using surface landmarks, this approach has since been substantially refined through the incorporation of nerve stimulation and, more recently, real-time ultrasound guidance [4]. Ultrasound visualization permits direct identification of neurovascular structures and confirmation of local anesthetic spread, thereby improving block success while reducing vascular puncture and other complications [5].

Ropivacaine, a long-acting amide local anesthetic, is preferred for peripheral nerve blockade because of its favorable therapeutic index and reduced cardiotoxic potential relative to bupivacaine [6]. Its clinical utility in regional anesthesia has been extensively validated across surgical settings [7]. Nevertheless, single-shot local anesthetic techniques remain constrained by a finite duration of action, prompting investigation of adjuvant agents to prolong analgesia [3].

Dexmedetomidine, a selective alpha-2 adrenergic agonist, has emerged as a promising perineural additive with documented capacity to potentiate and prolong local anesthetic blockade [8]. Its expanding clinical applications across anesthetic subspecialties have been well characterized [9]. Several studies have demonstrated significantly prolonged sensory and motor blockade when dexmedetomidine is combined with ropivacaine [10]. Fentanyl, a potent synthetic opioid acting on peripheral mu-opioid receptors, has similarly been proposed to expedite block onset [11], though other investigators have reported no meaningful improvement in block characteristics with its addition [12]. The mechanisms underlying peripheral opioid analgesia remain incompletely elucidated [13]. Given these conflicting findings, the present study was undertaken to compare dexmedetomidine and fentanyl as adjuvants to 0.75% ropivacaine in ultrasound-guided infraclavicular brachial plexus block, with the primary objective of comparing the onset and duration of sensory and motor blockade and the secondary objectives of comparing the duration of postoperative analgesia, block quality and success rate, perioperative hemodynamic parameters, and adverse effects. An observational design was chosen because the choice of adjuvant already varied according to established individual practice within the department, allowing comparison under real-world conditions without investigator-directed intervention.

## Materials and methods

Study design and setting

This was a prospective, comparative, observational study conducted in the Department of Anesthesiology at the Adichunchanagiri Institute of Medical Sciences (Belur, Karnataka, India), evaluating patients undergoing elective upper limb orthopedic surgery of the hand, wrist, forearm, and elbow performed under an ultrasound-guided infraclavicular brachial plexus block. Patients were categorized into one of two exposure groups - ropivacaine 0.75% with dexmedetomidine (Group A) or ropivacaine 0.75% with fentanyl (Group B) - according to the treating anesthesiologist's routine clinical practice at the time of block administration; the choice of adjuvant was not directed or controlled by the investigators. Patients were thereafter observed, and their block characteristics, hemodynamic parameters, and analgesic outcomes were recorded prospectively by the study team.

Study period

Patient enrollment and data collection were conducted over an 18-month period, extending from November 2016 to April 2018.

Ethics Committee approval

Approval was obtained from the Institutional Ethics Committee prior to commencement of the study (approval number: AIMS/IEC/1496/2016-17), and written informed consent was obtained from every participant after a detailed explanation of the observational procedures, in accordance with the Declaration of Helsinki. As this was an observational study, no experimental intervention outside standard clinical care was undertaken; only naturally occurring variation in adjuvant choice, arising from routine anesthetic practice, was observed and analyzed.

Inclusion criteria

Adult patients of either sex, aged between 18 and 65 years, classified as American Society of Anesthesiologists (ASA) physical status I or II, and scheduled for elective upper limb surgery under ultrasound-guided infraclavicular brachial plexus block with either dexmedetomidine or fentanyl combined with ropivacaine 0.75% as part of their routine anesthetic care, who provided written informed consent, were included.

Exclusion criteria

Patients younger than 18 years or older than 65 years; those with ASA physical status III or IV; those declining consent; those with known hypersensitivity to amide local anesthetics or the study adjuvants; patients with bleeding disorders or receiving anticoagulant therapy; those with local infection at the injection site, significant respiratory disease (including chronic obstructive pulmonary disease or hemidiaphragmatic palsy), or severe systemic (cardiac, hepatic, renal, neurological, psychiatric, or neurovascular) disease; pregnant or lactating women; morbidly obese patients; and those with pre-existing neurological deficit involving the operative limb were excluded.

Sample size estimation

Sample size was estimated using the standard formula for comparison of two independent sample means:

$N = \dfrac{\left(Z_{1-\alpha/2} + Z_{1-\beta}\right)^{2} \times 2 \times \sigma^{2}}{\left(\mu_{1} - \mu_{2}\right)^{2}}$

where $Z_{1-&alpha;/2}$ is the standard normal deviate corresponding to a two-sided type I error (alpha) of 0.05 (= 1.96), $Z_{1-\beta}$ is the standard normal deviate corresponding to 80% statistical power (= 0.84), σ is the pooled standard deviation of the primary outcome variable, and $(&mu;_1-&mu;_2)$ is the anticipated difference in means between the two groups.

Reference values were drawn from Esmaoglu et al. (2010), who studied 64 patients undergoing axillary brachial plexus block, comparing dexmedetomidine-supplemented levobupivacaine against plain levobupivacaine, and reported a mean duration of sensory blockade of 887 ± 66.2 minutes in the dexmedetomidine group versus 673.0 ± 73.8 minutes in the comparator group [14]. As the present study compares two active adjuvants rather than an adjuvant against a plain local anesthetic, a conservative anticipated between-group difference of 50 minutes was assumed, with a pooled standard deviation of 70.1 minutes derived from the reference study. Substituting these values in the formula above yielded a minimum requirement of 30 patients per group. Allowing for an anticipated 10% attrition or data-completeness deviation, a minimum of 30 patients per exposure group (total N = 60) was set as the enrollment target.

Sampling method

A consecutive sampling method was employed, whereby all eligible patients presenting for elective upper limb surgery during the study period were screened and enrolled in the order in which they presented and categorized into their respective exposure group according to the adjuvant used in their routine anesthetic care until the target sample size in each group was reached.

Data collection procedure

On the day preceding surgery, all enrolled patients underwent a standardized pre-anesthetic evaluation comprising detailed history, cardiorespiratory and airway examination, and relevant baseline investigations. Patients were kept nil per oral for a minimum of eight hours and received tablet ranitidine 150 mg and tablet alprazolam 0.5 mg as premedication on the night before surgery.

On the day of surgery, patients were positioned supine with the operative arm abducted to 90 degrees and the head turned to the opposite side. Intravenous access was secured, and continuous ECG, non-invasive blood pressure, and pulse oximetry monitoring were established, with baseline heart rate, systolic blood pressure, diastolic blood pressure, and mean arterial pressure recorded prior to block administration. The deltopectoral region was prepared with 5% povidone-iodine solution and draped in sterile fashion. Using a 5-8 MHz curvilinear ultrasound probe positioned in the infraclavicular fossa, the cords of the brachial plexus were identified in transverse cross-section relative to the axillary artery and vein, posterior to the pectoralis minor muscle. Following skin infiltration with 2% lignocaine, an 18-gauge Tuohy-tip needle was advanced in-plane under direct ultrasound visualization, and the study drug combination - ropivacaine 0.75% (30 mL) with either dexmedetomidine 50 micrograms (0.5 mL) plus 1.5 mL saline or fentanyl 75 micrograms (1.5 mL) plus 0.5 mL saline - was deposited around the plexus.

Sensory blockade was assessed by the pinprick method at five-minute intervals for up to 30 minutes across the C5-T1 dermatomes and graded as Grade 0 (sharp pin felt), Grade 1 (dull sensation), or Grade 2 (no sensation). Motor blockade was assessed concurrently using the modified Bromage scale for the upper extremity, graded as Grade 0 (full motor function), Grade 1 (movement of fingers only), or Grade 2 (complete motor block) [15]. Onset of sensory and motor blockade was defined as the time from completion of drug injection to first loss of pinprick sensation and first loss of motor power, respectively; complete blockade was defined as attainment of Grade 2 in each domain. Duration of sensory and motor blockade was recorded as the time from injection to return of pain sensation and motor function, respectively, and duration of postoperative analgesia was recorded as the time to the first request for rescue analgesia. Heart rate, respiratory rate, systolic blood pressure, diastolic blood pressure, mean arterial pressure, and peripheral oxygen saturation were recorded at baseline and at 5, 10, 15, 20, 25, and 30 minutes after drug administration and again at the end of the observation period. The occurrence of hypotension (fall in mean arterial pressure >20% of baseline), bradycardia (pulse rate ≤50/min), and other adverse effects, including respiratory depression, pneumothorax, hemothorax, nausea, vomiting, pruritus, and sedation, was recorded throughout, along with the management administered for each event.

Data analysis

Data were entered into a structured spreadsheet and analyzed using IBM SPSS Statistics for Windows, Version 26 (Released 2019; IBM Corp., Armonk, New York). Continuous variables, including age, weight, onset and duration of sensory and motor blockade, duration of postoperative analgesia, and hemodynamic and respiratory parameters, are expressed as mean ± standard deviation, with between-group comparisons performed using the independent-samples Student's t-test. Categorical variables, including sex distribution, ASA physical status, and grades of sensory and motor blockade, were expressed as frequencies and percentages and compared using the chi-square test. Time-point hemodynamic and respiratory data were compared between groups at each observation interval. A two-sided p-value of less than 0.05 was considered statistically significant for all analyses.

## Results

The two groups demonstrated comparable baseline characteristics in Table 1, thereby establishing therapeutic equipoise for the subsequent comparison of block dynamics. The mean age was 34.80 years with a standard deviation of 11.22 years in Group A, compared to 36.80 years with a standard deviation of 12.51 years in Group B (t = 0.652, p = 0.5171). Gender distribution was identical, comprising 22 male participants (73.33%) in each group (χ² = 0.000, p = 1.0000). Although mean body weight was notably lower in Group A at 61.13 kg (SD 9.38) than the 67.60 kg (SD 12.06) recorded in Group B, this represented the sole statistically significant demographic difference (t = 2.318, p = 0.0240).

**Table 1:** Baseline demographic and clinical characteristics of study participants undergoing ultrasound-guided infraclavicular brachial plexus block ^† ^Independent-samples t-test (df = 58) * Pearson chi-square test (df = 1) Data are presented as mean ± standard deviation (SD) for continuous variables and as n (%) for categorical variables. Statistical significance was set at p < 0.05. ASA: American Society of Anesthesiologists

Parameter	Group A: Dexmedetomidine (n = 30)	Group B: Fentanyl (n = 30)	Test Statistic	p-value
Age (years)	34.80 ± 11.22	36.80 ± 12.51	t = 0.652^†^	0.5171
Weight (kg)	61.13 ± 9.38	67.60 ± 12.06	t = 2.318^†^	0.0240
Gender, male — n (%)	22 (73.33%)	22 (73.33%)	χ² = 0.000*	1.0000
Gender, female — n (%)	8 (26.67%)	8 (26.67%)		
ASA physical status I — n (%)	24 (80.00%)	26 (86.67%)	χ² = 0.480*	0.4884
ASA physical status II — n (%)	6 (20.00%)	4 (13.33%)		

Analysis of block dynamics in Table 2 revealed a pronounced and consistent temporal advantage for the fentanyl adjuvant across every measured parameter. The mean onset of sensory blockade occurred at 8.45 minutes (SD 1.06) in Group A, compared to a considerably more rapid 1.83 minutes (SD 0.80) in Group B (t = 26.867, p = 0.0001). Similarly, motor blockade commenced at 11.27 minutes (SD 2.18) in Group A versus 2.87 minutes (SD 2.03) in Group B (t = 15.445, p = 0.0001). Complete sensory and motor anesthesia was likewise attained substantially earlier in Group B, with all differences reaching statistical significance, demonstrating that fentanyl expedites block establishment.

**Table 2:** Comparative onset and completion times of sensory and motor blockade between the two adjuvant groups Data are presented as mean ± standard deviation (SD) in minutes. Analysis was based on successfully blocked patients (Group A, n = 29; Group B, n = 29); onset of motor blockade was computed for all enrolled patients. Completion of motor blockade was assessed only in participants attaining Grade II motor block (Group A, n = 25; Group B, n = 16).

Parameter	Group A: Dexmedetomidine	Group B: Fentanyl	Independent-Samples t-test	p-value
Onset of sensory blockade (min)	8.45 ± 1.06	1.83 ± 0.80	26.867	0.0001
Onset of motor blockade (min)	11.27 ± 2.18	2.87 ± 2.03	15.445	0.0001
Complete sensory blockade (min)	12.90 ± 2.06	5.90 ± 2.38	11.977	0.0001
Complete motor blockade (min)	20.12 ± 4.99	7.06 ± 2.32	9.785	0.0001

Evaluation of block quality in Table 3 revealed divergent profiles for the sensory and motor components. Complete (Grade II) sensory blockade was achieved in 29 participants (96.67%) in each group, with the solitary failure per group being attributable to dermatomal sparing; consequently, no statistically significant difference was observed (χ² = 0.000, p = 1.0000). Conversely, complete motor blockade was established in 24 participants (80.00%) in Group A compared with only 16 (53.33%) in Group B, whereas partial (Grade I) motor blockade was correspondingly more frequent in Group B at 14 participants (46.67%). This disparity in motor block density proved statistically significant (χ² = 4.800, p = 0.0285), indicating superior motor blockade with dexmedetomidine. Failures in both groups were attributed to incomplete circumferential spread of local anesthetic, likely reflecting technical or anatomical variability, and both patients were converted to general anesthesia.

**Table 3:** Comparative quality of sensory and motor blockade between the two adjuvant groups Data are presented as n (%). Grade 0 denotes absent blockade (block failure); Grade I denotes partial motor blockade; Grade II denotes complete blockade.

Parameter	Group A: Dexmedetomidine (n = 30)	Group B: Fentanyl (n = 30)	Pearson Chi-Square Test	p-value
Sensory block, Grade II (complete) — n (%)	29 (96.67%)	29 (96.67%)	0.000	1.0000
Sensory block, Grade 0 (failed) — n (%)	1 (3.33%)	1 (3.33%)		
Motor block, Grade II (complete) — n (%)	24 (80.00%)	16 (53.33%)	4.800	0.0285
Motor block, Grade I (partial) — n (%)	6 (20.00%)	14 (46.67%)		

The duration of every block characteristic was substantially prolonged by the dexmedetomidine adjuvant, representing the principal therapeutic finding of the investigation as shown in Table 4. The mean duration of sensory blockade was 883.17 minutes (SD 66.27) in Group A, more than double the 404.00 minutes (SD 95.85) observed in Group B (t = 22.144, p < 0.0001). Motor blockade endured for a mean of 819.07 minutes (SD 58.13) in Group A compared with 380.38 minutes (SD 118.59) in Group B (t = 17.887, p < 0.0001). Furthermore, postoperative analgesia was sustained for 977.79 minutes (SD 66.90) in Group A versus 492.66 minutes (SD 106.14) in Group B (t = 20.824, p < 0.0001), a considerable and clinically meaningful extension.

**Table 4:** Comparative duration of sensory blockade, motor blockade, and postoperative analgesia between the two adjuvant groups Data are presented as mean ± standard deviation (SD). Statistical significance was set at p < 0.05.

Parameter	Group A: Dexmedetomidine (n = 30)	Group B: Fentanyl (n = 30)	Independent t-test	p-value
Duration of sensory blockade (min)	883.17 ± 66.27	404.00 ± 95.85	22.144	0.0001
Duration of motor blockade (min)	819.07 ± 58.13	380.38 ± 118.59	17.887	0.0001
Duration of postoperative analgesia (min)	977.79 ± 66.90	492.66 ± 106.14	20.824	0.0001

Perioperative respiratory stability was preserved throughout the observation period in both cohorts, as shown in Table 5. The mean pulse rate remained comparable at baseline, measuring 78.23 beats/min (SD 11.91) in Group A and 81.80 beats/min (SD 11.31) in Group B (t = 1.189, p = 0.2391); however, a transient yet statistically significant elevation emerged in Group B at 5 and 10 minutes, reaching 87.60 beats/min (SD 10.82; t = 2.842, p = 0.0062) and 86.27 beats/min (SD 10.45; t = 2.335, p = 0.0230), respectively, before converging thereafter. Conversely, respiratory rate demonstrated no statistically significant difference at any time point, confirming the absence of clinically relevant respiratory depression with either adjuvant.

**Table 5:** Comparative perioperative pulse rate and respiratory rate between the two adjuvant groups across time points Data are presented as mean ± standard deviation (SD). Statistical significance was set at p < 0.05.

Time Point	Group A: Dexmedetomidine (n = 30)	Group B: Fentanyl (n = 30)	Independent-Samples t-test (df = 58)	p-value
Pulse rate (beats/min)				
Baseline	78.23 ± 11.91	81.80 ± 11.31	1.189	0.2391
5 minutes	78.77 ± 13.14	87.60 ± 10.82	2.842	0.0062
10 minutes	79.10 ± 13.17	86.27 ± 10.45	2.335	0.0230
15 minutes	79.30 ± 13.39	83.50 ± 10.82	1.337	0.1866
20 minutes	79.63 ± 12.31	83.80 ± 11.14	1.374	0.1746
25 minutes	79.77 ± 12.65	84.33 ± 11.29	1.475	0.1455
30 minutes	81.13 ± 13.14	87.00 ± 13.28	1.720	0.0908
End of study	81.00 ± 12.66	86.50 ± 10.42	1.837	0.0713
Respiratory rate (cycles/min)				
Baseline	13.67 ± 1.67	14.00 ± 1.80	0.744	0.4599
5 minutes	13.40 ± 1.38	13.70 ± 1.53	0.796	0.4291
10 minutes	13.50 ± 1.11	13.33 ± 1.42	0.507	0.6144
15 minutes	13.57 ± 1.61	14.03 ± 1.43	1.188	0.2398
20 minutes	13.23 ± 1.25	13.53 ± 1.25	0.928	0.3570
25 minutes	13.33 ± 1.37	13.43 ± 1.48	0.271	0.7870
30 minutes	13.63 ± 1.50	13.43 ± 1.17	0.578	0.5658
End of study	13.73 ± 1.53	13.87 ± 1.36	0.357	0.7224

Divergent hemodynamic trajectories were observed between the adjuvant groups, as shown in Table 6. Systolic pressure was equivalent at baseline (123.77 ± 11.94 mmHg versus 121.80 ± 14.07 mmHg; t = 0.584, p = 0.5616) but became significantly higher in the fentanyl group at every subsequent interval, peaking at 142.67 mmHg (SD 21.11) at 20 minutes (t = 3.807, p = 0.0003). Diastolic pressure was conversely and consistently higher in the dexmedetomidine group throughout, with statistically significant differences at all time points (p-values ranging from 0.0362 to 0.0001). Mean arterial pressure differed significantly only at baseline (t = 2.555, p = 0.0133), reflecting the counterbalancing systolic and diastolic shifts and the recognized sympatholytic action of dexmedetomidine.

**Table 6:** Comparative perioperative systolic, diastolic, and mean arterial pressures between the two adjuvant groups across time points Data are presented as mean ± standard deviation (SD). Statistical significance was set at p < 0.05.

Time Point	Group A: Dexmedetomidine (n = 30)	Group B: Fentanyl (n = 30)	Independent-samples t-test (df = 58)	p-value
Systolic blood pressure (mmHg)				
Baseline	123.77 ± 11.94	121.80 ± 14.07	0.584	0.5616
5 minutes	126.47 ± 11.87	138.80 ± 17.33	3.216	0.0021
10 minutes	125.27 ± 12.13	139.27 ± 18.73	3.436	0.0011
15 minutes	125.60 ± 11.71	141.83 ± 18.75	4.021	0.0002
20 minutes	125.90 ± 11.67	142.67 ± 21.11	3.807	0.0003
25 minutes	124.80 ± 11.42	140.27 ± 20.56	3.602	0.0007
30 minutes	125.67 ± 10.90	141.23 ± 23.06	3.342	0.0015
End of study	126.73 ± 10.51	140.77 ± 21.07	3.265	0.0018
Diastolic blood pressure (mmHg)				
Baseline	83.10 ± 6.97	75.93 ± 9.76	3.272	0.0018
5 minutes	83.83 ± 7.34	74.40 ± 12.43	3.579	0.0007
10 minutes	82.03 ± 7.70	75.17 ± 9.79	3.019	0.0038
15 minutes	81.67 ± 7.17	75.83 ± 13.07	2.144	0.0362
20 minutes	81.93 ± 7.60	75.83 ± 12.76	2.250	0.0283
25 minutes	82.33 ± 4.26	74.53 ± 14.28	2.867	0.0058
30 minutes	83.37 ± 5.35	73.67 ± 11.59	4.163	0.0001
End of study	84.50 ± 5.24	75.33 ± 10.43	4.302	0.0001
Mean arterial pressure (mmHg)				
Baseline	96.66 ± 6.00	91.12 ± 10.24	2.555	0.0133
5 minutes	98.00 ± 6.66	95.86 ± 12.38	0.832	0.4087
10 minutes	96.47 ± 6.90	96.53 ± 10.91	0.027	0.9786
15 minutes	96.33 ± 6.32	97.63 ± 13.17	0.487	0.6284
20 minutes	96.43 ± 6.23	98.11 ± 13.85	0.604	0.5481
25 minutes	96.30 ± 4.79	96.44 ± 15.26	0.048	0.9617
30 minutes	97.57 ± 5.64	96.19 ± 13.71	0.510	0.6118
End of study	98.60 ± 5.34	97.20 ± 12.64	0.559	0.5785

Peripheral oxygen saturation remained clinically optimal throughout the perioperative period in both cohorts, as shown in Table 7. Although a statistically significant difference was detected at baseline, with a mean of 99.70% (SD 0.60) in Group A and 99.97% (SD 0.18) in Group B (t = 2.343, p = 0.0226), this fractional variance was devoid of clinical relevance; from 10 minutes onward, both groups maintained a uniform saturation of 100.00%, precluding further comparison. No adverse events, including hypotension, bradycardia, respiratory depression, pneumothorax, or emesis, were observed with either adjuvant, and the solitary block failure per group was managed uneventfully with general anesthesia, establishing a comparable and favorable safety profile for both regimens.

**Table 7:** Comparative perioperative peripheral oxygen saturation and adverse events between the two adjuvant groups across time points Data are presented as peripheral oxygen saturation (SpO₂, %) as mean ± standard deviation (SD). No episodes of hypotension, bradycardia, respiratory depression, pneumothorax, nausea, or vomiting were recorded in either group. Statistical significance was set at p < 0.05.

Time Point	Group A: Dexmedetomidine (n = 30)	Group B: Fentanyl (n = 30)	Independent-Samples t-test	p-value
Baseline	99.70 ± 0.60	99.97 ± 0.18	2.343	0.0226
5 minutes	99.77 ± 0.68	100.00 ± 0.00	1.882	0.0648
10 minutes	100.00 ± 0.00	100.00 ± 0.00	—	—
15 minutes	100.00 ± 0.00	100.00 ± 0.00	—	—
20 minutes	100.00 ± 0.00	100.00 ± 0.00	—	—
25 minutes	100.00 ± 0.00	100.00 ± 0.00	—	—
30 minutes	100.00 ± 0.00	100.00 ± 0.00	—	—
End of study	100.00 ± 0.00	100.00 ± 0.00	—	—

## Discussion

The present observational study compared dexmedetomidine and fentanyl as adjuvants to 0.75% ropivacaine in ultrasound-guided infraclavicular brachial plexus block, revealing a markedly faster onset of both sensory and motor blockade with fentanyl, with sensory blockade commencing at 1.83 minutes (SD 0.80) in the fentanyl group compared with 8.45 minutes (SD 1.06) in the dexmedetomidine group and motor blockade commencing at 2.87 minutes (SD 2.03) versus 11.27 minutes (SD 2.18), respectively (p = 0.0001 for both). This finding is congruent with Moharari et al. (2010), who reported that fentanyl supplementation expedited onset of both sensory and motor blockade in interscalene lidocaine anesthesia, attributing this effect to peripheral opioid-receptor-mediated potentiation of local anesthetic conduction blockade [16]. Chavan et al. (2011) similarly observed accelerated analgesic onset with fentanyl added to local anesthetic during brachial plexus block [17]. However, this observation contrasts with Nishikawa et al. (2000), who found that fentanyl, while improving analgesic quality, in fact prolonged rather than shortened the onset of axillary brachial plexus block, a discrepancy possibly attributable to differences in block approach, local anesthetic agent, or opioid dose [11]. Fanelli et al. (2001) likewise reported no meaningful improvement in onset characteristics with fentanyl in axillary block, underscoring the inconsistent peripheral opioid effects across anatomical approaches and patient populations [12].

Despite its delayed onset in the present cohort, dexmedetomidine demonstrated a substantially superior duration of both sensory and motor blockade and postoperative analgesia. Duration of sensory blockade was 883.17 minutes (SD 66.27) in the dexmedetomidine group compared with 404.00 minutes (SD 95.85) in the fentanyl group; motor blockade duration was 819.07 minutes (SD 58.13) versus 380.38 minutes (SD 118.59); and postoperative analgesia extended to 977.79 minutes (SD 66.90) compared with 492.66 minutes (SD 106.14) (p = 0.0001 for all). These findings closely parallel those of Esmaoglu et al. (2010), who reported that dexmedetomidine added to levobupivacaine in an axillary block prolonged sensory blockade to 887 minutes (SD 66.2) and postoperative analgesia to 1008.7 minutes (SD 164.0), values strikingly similar in magnitude to those obtained here [14]. Comparable prolongation with dexmedetomidine has also been documented by Biswas et al. (2014) in a supraclavicular block with levobupivacaine, by Gurajala et al. (2015) using perineural dexmedetomidine with ropivacaine, and by Marhofer et al. (2013) in a volunteer study demonstrating prolonged peripheral nerve block duration with dexmedetomidine-supplemented ropivacaine [10,18,19]. This magnitude of prolongation is further consistent with Ammar and Mahmoud (2012), who reported significantly extended analgesic duration with dexmedetomidine-supplemented bupivacaine in a single-injection infraclavicular block, and with Rashmi and Komala (2017), who documented similarly prolonged sensory and motor blockade with dexmedetomidine as an adjuvant to 0.75% ropivacaine in an interscalene block [20,21]. This consistency across anatomical approaches and local anesthetic combinations reinforces the pharmacological plausibility of the alpha-2 adrenergic mechanism described by Grewal (2011) and Kaur (2011), whereby dexmedetomidine potentiates local anesthetic action through hyperpolarization-activated cation current inhibition in peripheral nerve fibers alongside central sympatholytic effects [8,22].

Beyond temporal parameters, the present study revealed a qualitative advantage of dexmedetomidine in motor block density. Complete (Grade II) motor blockade was achieved in 24 patients (80.00%) in the dexmedetomidine group compared with 16 patients (53.33%) in the fentanyl group, while partial (Grade I) blockade was more frequent with fentanyl, occurring in 14 patients (46.67%) versus six patients (20.00%) with dexmedetomidine (p=0.0285). This aligns with Kumar et al. (2017), who reported denser, more complete motor blockade with dexmedetomidine compared with fentanyl as bupivacaine adjuvants in supraclavicular block [23]. In contrast, sensory blockade quality was statistically indistinguishable between groups, with complete sensory blockade achieved in 29 patients (96.67%) in each group, indicating that fentanyl's accelerated onset does not compromise ultimate sensory anesthesia depth, consistent with Rajkhowa et al. (2016), who reported comparable sensory block success rates between opioid-supplemented and non-opioid regimens [24].

Hemodynamic analysis revealed divergent trends between adjuvants. Systolic blood pressure was significantly higher in the fentanyl group throughout, peaking at 142.67 mmHg (SD 21.11) at 20 minutes compared with 125.90 mmHg (SD 11.67) in the dexmedetomidine group (p=0.0003), while diastolic pressure was consistently higher in the dexmedetomidine group at every time point (p-values ranging from 0.0362 to 0.0001). Pulse rate showed a transient elevation in the fentanyl group at five minutes, reaching 87.60 beats/min (SD 10.82) versus 78.77 beats/min (SD 13.14) in the dexmedetomidine group (p=0.0062). These observations support the sympatholytic property of dexmedetomidine described by Grewal (2011) and the modest bradycardic and hypotensive tendencies reviewed by Mason (2011), although in the present cohort these effects remained clinically inconsequential and required no pharmacological correction [8,9]. No hypotension, bradycardia, respiratory depression, pneumothorax, or emesis occurred in either group, and the single block failure per group was managed uneventfully with general anesthesia - a safety profile consistent with the low complication rates reported by Chin et al. (2013) and Sandhu and Capan (2002) for ultrasound-guided infraclavicular approaches and with the comparative safety data of Mirkheshti et al. (2014) evaluating dexmedetomidine against ketorolac as an infraclavicular adjuvant [4,25,26].

The reliability of ultrasound guidance observed in this study further corroborates the technical descriptions of Ootaki et al. (2000) and Zaragoza-Lemus et al. (2015), who established ultrasound visualization as superior to landmark-based techniques for catheter placement accuracy [3,27]. The choice of ropivacaine 0.75% as a base local anesthetic is well supported by its favorable safety index relative to bupivacaine, as described by McClure (1996) and Kuthiala and Chaudhary (2011), and by its comparative analgesic potency demonstrated by Borgeat et al. (2001) [6,7,28]. Collectively, the divergent onset-versus-duration profiles observed here, situated within a substantial body of concordant literature, suggest that adjuvant selection should be individualized to the clinical priority at hand - rapid surgical readiness favoring fentanyl or prolonged postoperative analgesia favoring dexmedetomidine - echoing the nuanced conclusion of Thakkar et al. (2018) regarding dexmedetomidine's role in optimizing analgesic quality in infraclavicular block [29].

Clinical significance

These findings carry direct implications for perioperative pain management in upper limb surgery. Because dexmedetomidine nearly doubled the duration of motor and sensory blockade and postoperative analgesia compared with fentanyl, its use as a ropivacaine adjuvant in ultrasound-guided infraclavicular block may substantially reduce postoperative opioid requirements, consistent with the reduced systemic opioid reliance associated with peripheral nerve blockade described by Richman et al. (2006) [1]. Conversely, fentanyl's rapid onset may be advantageous in settings demanding expedited surgical readiness, such as high-turnover ambulatory surgical units, supported by Harrison et al. (2015) in their comparison of infraclavicular and supraclavicular perineural catheter techniques [30]. The comparable and favorable safety profile of both adjuvants, without respiratory depression or hemodynamic compromise, supports their continued use in both ambulatory and inpatient settings, reinforcing the broader safety consensus articulated by Marhofer et al. (2010) regarding ultrasound-guided regional anesthesia [31].

Strength of the study

Key strengths include the prospective design with standardized, validated assessment tools for sensory and motor blockade - the pinprick method and modified Bromage scale, respectively - applied consistently across both exposure groups. Consecutive enrollment of all eligible patients presenting during the study period reduced the potential for selection bias inherent to non-random exposure grouping. Ultrasound guidance ensured anatomically accurate needle and catheter placement, supporting the reliability of block outcomes. The sample size, calculated a priori from comparable published data, provided adequate power to detect clinically meaningful between-group differences. Comprehensive assessment of both efficacy parameters (onset, duration, and quality of block) and safety parameters (hemodynamic stability and adverse events) across multiple time points further strengthens the internal validity and clinical applicability of the findings.

Limitations

As an observational design, exposure group allocation was not subject to strict investigator-controlled randomization, introducing potential selection bias despite comparable baseline demographics. The single-center design may limit generalizability to other institutional or patient populations. While adequately powered for the primary duration outcomes, the sample size may have been insufficient to detect smaller but clinically relevant differences in uncommon adverse events. Postoperative analgesia was assessed only until the first rescue analgesic request, without extended follow-up into later postoperative recovery or chronic pain outcomes. Cost-effectiveness and patient satisfaction, both clinically relevant to adjuvant selection, were not formally assessed and warrant further study. Outcome assessment was performed by the treating anesthesiologists rather than an independent blinded observer, which may have introduced observer bias in the subjectively rated block parameters, and residual confounding by unmeasured variables such as surgical type and duration cannot be excluded.

Recommendations

Future multicenter studies with larger samples are recommended to validate these findings across diverse populations and surgical settings. Dose-ranging studies comparing varying dexmedetomidine and fentanyl concentrations may help identify optimal dosing balancing rapid onset with prolonged analgesia. Incorporating patient-reported satisfaction, postoperative opioid consumption, and cost-effectiveness analyses would further inform adjuvant selection. Long-term follow-up evaluating chronic postsurgical pain outcomes following brachial plexus blockade with these adjuvants is also warranted.

## Conclusions

This observational study demonstrated that dexmedetomidine and fentanyl, used as adjuvants to 0.75% ropivacaine in ultrasound-guided infraclavicular brachial plexus block, exhibit distinct and clinically complementary pharmacological profiles. Fentanyl produced a markedly faster onset of sensory and motor blockade, favoring scenarios prioritizing rapid surgical readiness. Dexmedetomidine conferred a substantially longer duration of sensory blockade, motor blockade, and postoperative analgesia, alongside a denser, more complete motor block, supporting its preferential use where extended postoperative pain control is the primary clinical goal. Both adjuvants demonstrated a favorable and broadly comparable safety profile, with no significant adverse events, stable oxygen saturation, and only minor, clinically inconsequential hemodynamic fluctuations throughout the perioperative period. These findings suggest that the choice between dexmedetomidine and fentanyl as ropivacaine adjuvants should be individualized to the specific clinical priority of the surgical scenario rather than applying a uniform approach. Further multicenter, adequately powered studies are warranted to corroborate these findings, refine optimal adjuvant dosing, and clarify their comparative cost-effectiveness and impact on patient-centered outcomes in upper limb regional anesthesia.

## References

[1] Richman JM, Liu SS, Courpas G (2006). Does continuous peripheral nerve block provide superior pain control to opioids? A meta-analysis. Anesth Analg.

[2] Kaye AD, Allampalli V, Fisher P (2021). Supraclavicular vs. infraclavicular brachial plexus nerve blocks: clinical, pharmacological, and anatomical considerations. Anesth Pain Med.

[3] Zaragoza-Lemus G, Hernández-Gasca V, Espinosa-Gutiérrez A (2015). Ultrasound-guided continuous infraclavicular block for hand surgery: technical report arm position for perineural catheter placement (Article in Spanish). Cir Cir.

[4] Chin KJ, Alakkad H, Adhikary SD, Singh M (2013). Infraclavicular brachial plexus block for regional anaesthesia of the lower arm. Cochrane Database Syst Rev.

[5] Chan VW, Perlas A, Rawson R, Odukoya O (2003). Ultrasound-guided supraclavicular brachial plexus block. Anesth Analg.

[6] McClure JH (1996). Ropivacaine. Br J Anaesth.

[7] Kuthiala G, Chaudhary G (2011). Ropivacaine: a review of its pharmacology and clinical use. Indian J Anaesth.

[8] Grewal A (2011). Dexmedetomidine: new avenues. J Anaesthesiol Clin Pharmacol.

[9] Mason KP, Lerman J (2011). Dexmedetomidine in children: current knowledge and future applications. Anesth Analg.

[10] Marhofer D, Kettner SC, Marhofer P, Pils S, Weber M, Zeitlinger M (2013). Dexmedetomidine as an adjuvant to ropivacaine prolongs peripheral nerve block: a volunteer study. Br J Anaesth.

[11] Nishikawa K, Kanaya N, Nakayama M, Igarashi M, Tsunoda K, Namiki A (2000). Fentanyl improves analgesia but prolongs the onset of axillary brachial plexus block by peripheral mechanism. Anesth Analg.

[12] Fanelli G, Casati A, Magistris L (2001). Fentanyl does not improve the nerve block characteristics of axillary brachial plexus anaesthesia performed with ropivacaine. Acta Anaesthesiol Scand.

[13] Pardo MC (2023). Miller’s Basics of Anesthesia, Eighth Edition. https://evolve.elsevier.com/cs/product/9780323796774?role=student.

[14] Esmaoglu A, Yegenoglu F, Akin A, Turk CY (2010). Dexmedetomidine added to levobupivacaine prolongs axillary brachial plexus block. Anesth Analg.

[15] Singh DR, Mohamed H, Krishnaveni N, Nag K (2017). Evaluating the efficacy of tramadol as an adjuvant to intrathecal isobaric levobupivacaine for elective infraumbilical surgeries. Anesth Essays Res.

[16] Moharari R, Sadeghi J, Khajavi M, Davari M, Mojtahedzadeh M (2010). Fentanyl supplement expedites the onset time of sensory and motor blocking in interscalene lidocaine anesthesia. Daru.

[17] Chavan SG, Koshire AR, Panbude P (2011). Effect of addition of fentanyl to local anesthetic in brachial plexus block on duration of analgesia. Anesth Essays Res.

[18] Biswas S, Das RK, Mukherjee G, Ghose T (2014). Dexmedetomidine an adjuvant to levobupivacaine in supraclavicular brachial plexus block: a randomized double blind prospective study. Ethiop J Health Sci.

[19] Gurajala I, Thipparampall AK, Durga P, Gopinath R (2015). Effect of perineural dexmedetomidine on the quality of supraclavicular brachial plexus block with 0.5% ropivacaine and its interaction with general anaesthesia. Indian J Anaesth.

[20] Ammar AS, Mahmoud KM (2012). Ultrasound-guided single injection infraclavicular brachial plexus block using bupivacaine alone or combined with dexmedetomidine for pain control in upper limb surgery: a prospective randomized controlled trial. Saudi J Anaesth.

[21] Rashmi HD, Komala HK (2017). Effect of dexmedetomidine as an adjuvant to 0.75% ropivacaine in interscalene brachial plexus block using nerve stimulator: a prospective, randomized double-blind study. Anesth Essays Res.

[22] Kaur M, Singh PM (2011). Current role of dexmedetomidine in clinical anesthesia and intensive care. Anesth Essays Res.

[23] Kumar B, Gadre V, Basavaraj P (2017). Comparison of dexmedetomidine and fentanyl as an adjuvant to 0.25 % bupivacaine in supraclavicular brachial plexus block. JMSCR.

[24] Rajkhowa T, Das N, Parua S, Kundu R (2016). Fentanyl as an adjuvant for brachial plexus block: a randomized comparative study. Int J Clin Trials.

[25] Mirkheshti A, Saadatniaki A, Salimi A, Manafi Rasi A, Memary E, Yahyaei H (2014). Effects of dexmedetomidine versus ketorolac as local anesthetic adjuvants on the onset and duration of infraclavicular brachial plexus block. Anesth Pain Med.

[26] Sandhu NS, Capan LM (2002). Ultrasound-guided infraclavicular brachial plexus block. Br J Anaesth.

[27] Ootaki C, Hayashi H, Amano M (2000). Ultrasound-guided infraclavicular brachial plexus block: an alternative technique to anatomical landmark-guided approaches. Reg Anesth Pain Med.

[28] Borgeat A, Kalberer F, Jacob H, Ruetsch YA, Gerber C (2001). Patient-controlled interscalene analgesia with ropivacaine 0.2% versus bupivacaine 0.15% after major open shoulder surgery: the effects on hand motor function. Anesth Analg.

[29] Thakkar F, Parmar V, Oza V, Sarathy S (2018). Infraclavicular brachial plexus block in upper limb surgeries with local anaesthetic agent and dexmedetomidine as adjuvant for improving quality of analgesia. J Anesthesiol Clin Sci.

[30] Harrison TK, Kim TE, Howard SK (2015). Comparative effectiveness of infraclavicular and supraclavicular perineural catheters for ultrasound-guided through-the-catheter bolus anesthesia. J Ultrasound Med.

[31] Marhofer P, Harrop-Griffiths W, Kettner SC, Kirchmair L (2010). Fifteen years of ultrasound guidance in regional anaesthesia: part 1. Br J Anaesth.

